# Explaining institutional variation in healthcare service quality: evidence from public hospitals in KwaZulu-Natal, South Africa

**DOI:** 10.1108/IJHCQA-05-2026-0129

**Published:** 2026-11-02

**Authors:** Zinhle P. Khumalo

**Affiliations:** Department of Public Management and Economics, Durban University of Technology–ML Sultan Campus, Durban, South Africa

**Keywords:** Service quality, Institutional theory, Public hospitals, Human capital development, Service Handling Practices, KwaZulu-Natal, SDG 3, Agenda 2063

## Abstract

**Purpose:**

This study explains variation in healthcare service quality through an institutional and process-oriented lens rather than treating quality as a simple statistical comparison between hospitals. Drawing on institutional theory and Donabedian's structure-process-outcome logic, it examines whether Human Capital Development Activities (HCDA), Research Attitudes and Work Skills (RAWS) and workforce experience are associated with Service Handling Practices (SHP) in selected public hospitals in KwaZulu-Natal, South Africa. The study is positioned within Sustainable Development Goal 3, particularly target 3.8 on access to quality essential healthcare services, and African Union Agenda 2063 Goal 3 on healthy and well-nourished citizens.

**Design/methodology/approach:**

A quantitative cross-sectional survey design was used. The respondent-level dataset contained 238 questionnaires. The analysis uses three measured constructs: HCDA (12 items), SHP (9 items) and RAWS (13 items), each assessed on 5-point Likert-type scales. Construct adequacy was examined using Kaiser–Meyer–Olkin statistics, Bartlett's tests, exploratory component analysis and Cronbach's alpha. Work-experience differences were assessed using one-way ANOVA with effect sizes. Multiple regression estimated SHP as the process-oriented service-quality outcome with HCDA and RAWS as predictors. Heteroscedasticity-consistent HC3 standard errors were used after diagnostic testing.

**Findings:**

The regression was statistically significant overall, F(2, 231) = 4.35, *p* = 0.014, but explanatory power was modest (*R*^2^ = 0.036; adjusted *R*^2^ = 0.028). HCDA (B = 0.080, robust *p* = 0.376) and RAWS (B = 0.134, robust *p* = 0.152) were not independently significant after mutual adjustment. Work experience differed significantly across HCDA groups (F = 2.221, *p* = 0.042), but the practical effect was small (ω^2^ = 0.030); differences in SHP and RAWS were not significant. The results therefore do not support a simple claim that tenure or a single institutional attribute independently determines service quality. Rather, they refer to a more comprehensive institutional setting where governance procedures, organisational assistance, learning, coordination and service procedures all have a role to play.

**Research limitations/implications:**

Cross-sectional, self-reported; does not suggest causality. Unfortunately, the de-identified analytic data set did not contain sufficient detail to assure reliable identification of hospitals, nor did it contain profession-specific respondent labels which would have enabled multilevel hospital effects and profession-specific comparisons. The SHP scale is a process-oriented scale and exploratory analysis indicates that the scales in the survey are multidimensional. Future studies should employ hospital-based longitudinal data and profession-stratified sampling in addition to confirmatory measurement models and objective clinical and patient-experience outcomes.

**Practical implications:**

Managers must not put their trust in the age of employment of personnel as a proxy for organisational capacity. Continuous professional development should be embedded in supervision, patient-flow redesign, communication routines, accountability systems and resource-management processes. Quality-improvement dashboards should therefore combine workforce, process, governance and patient-experience indicators.

**Social implications:**

Strengthening the organisational processes through which public hospitals convert staff capability and resources into reliable care supports the quality dimension of universal health coverage and the health-system aspirations of SDG 3 and Agenda 2063.

**Originality/value:**

The study contributes by aligning measurable workforce and institutional conditions with a process-oriented quality outcome and by combining institutional theory with Donabedian's process logic. It shows that workforce development and work skills are enabling but insufficient conditions for service quality, supporting a systems-based rather than single-factor explanation of public-hospital performance.

## 1. Introduction

Quality health services are central to contemporary health-system performance because access alone does not guarantee effective, safe, timely or people-centred care. The global quality agenda therefore treats service quality as an institutional property of health systems as well as an outcome of individual clinical practice (World Health Organization, Organisation for Economic Co-operation and Development and World Bank, 2018; Kruk *et al.*, 2018). This concern is directly connected to Sustainable Development Goal (SDG) 3.8, which calls for universal health coverage that includes access to quality essential healthcare services, and to African Union (AU) Agenda 2063 Goal 3, which envisages healthy and well-nourished citizens through expanded access to quality health care (United Nations, 2015; African Union, 2015).

Public hospitals provide a useful setting for examining this problem because service quality depends on more than the availability of beds, equipment or staff. It also depends on whether institutions coordinate people and resources through effective routines, learning systems, accountability arrangements and patient-facing processes. Donabedian (1988) structure-process-outcome model makes this distinction explicit: structural inputs create capacity, but quality is realised through processes that convert those inputs into care. Institutional theory adds a complementary explanation by focusing on the formal rules, organisational routines, norms and taken-for-granted practices that shape how organisations act under similar policy environments (Meyer and Rowan, 1977; DiMaggio and Powell, 1983; Scott, 2014).

The international literature increasingly recognises that apparently similar health facilities can produce different outcomes because organisational processes condition how resources are used. In high-quality health systems, competent care depends on governance, learning, workforce support and reliable service processes rather than infrastructure alone (Kruk *et al.*, 2018). Recent African evidence reinforces this institutional perspective. Kyambade *et al.* (2026), using institutional theory in Uganda's health and sanitation sector, show that fragmented governance, weak accountability and limited local ownership can undermine performance despite policies and investment. In Tenza *et al.* (2024) demonstrate that pharmaceutical distribution performance depends on operational and information-management dynamics, while Mtengule *et al.* (2026) show how institutional adoption of e-tendering shaped continuity in healthcare procurement during the COVID-19 period. These studies illustrate that quality-related performance is produced through organisational systems, not through resources in isolation.

South Africa faces the same analytical challenge. National evidence shows that the practice environment is associated with nurses' perceptions of quality and patient safety across different public-hospital levels (Tenza *et al.*, 2024). KwaZulu-Natal-specific research also demonstrates variation in the technical efficiency of public district hospitals and identifies staffing mix, utilisation and expenditure as factors associated with performance (Babalola *et al.*, 2022). These findings indicate that institutional capacity and service processes deserve attention alongside patient satisfaction and resource counts. The KwaZulu-Natal Department of Health operates a heterogeneous public-hospital network that includes district, regional, tertiary and specialised facilities, making the province an appropriate context for examining how organisational conditions relate to service processes (KwaZulu-Natal Department of Health, 2025).

Existing studies frequently operationalise healthcare service quality through broad determinant categories such as administration, responsiveness, communication and infrastructure. Such categories are analytically useful only when they are represented by observable and sufficiently reliable measures. Thus, this study uses an empirical model consisting of three constructs that remained at the Respondent level: Human Capital Development Activities (HCDA), Service Handling Practices (SHP) and Research Attitudes and Work Skills (RAWS). SHP is considered a process-oriented indicator of service quality; HCDA and RAWS are indicators of organisational and workforce conditions which can enable effective service handling. Work experience is looked at as an individual characteristic that can have an effect irrespective of institutional support.

Using this, the following questions are asked: (1) Is HCDA related to SHP and RAWS after mutual adjustment? (2) Does work experience have any relationship with HCDA, SHP and RAWS? and (3) How these relationships are explained with the help of institutional theory and the Donabedian model? This does not, therefore, mean that a particular resource or population factor mechanically predisposes for quality. Rather, it is an incremental hypothesis: The ability of institutions is expressed in the interplay between organisational and workforce processes, and individual experience is not likely to lead to better service delivery unless it is converted to ability through supportive organisational processes.

## 2. Literature review and theoretical framework

### 2.1 Service quality as an institutional and process outcome

Health systems can have many different aspects of quality. Donabedian (1988) identified three parameters, providing an underpinning to analyse the way organisational inputs are translated into the provision of actual care: structure, process and outcome. Effective and safe care, and care that is people-centred, timely, equitable, integrated and efficient, are also important factors included in today's quality frameworks (World Health Organization, Organisation for Economic Co-operation and Development and World Bank, 2018). SHP are conceptually situated in the process domain: they are not necessarily related to clinical outcomes like mortality or readmission but to how services are organised and enacted.

At the public hospitals, it is important, for good facilities will not necessarily give good systems. Even though a hospital can have the necessary expertise and policies, it can still show poor coordination and have ineffective service routines or inconsistent supervision. The quality for the patient is therefore related to institutional reliability: the routine of appropriate action being supported and accountable. This is in line with recent South African research, which found that the practice environment and organisational context are correlated with the perceived quality and safety (Tenza *et al.*, 2024).

### 2.2 Institutional theory

Institutional theory is concerned with the formal rules, norms and common cognitive elements that emerge in an organisation and impact on the functioning of the organisation (Meyer and Rowan, 1977; DiMaggio and Powell, 1983; Scott, 2014). Although there are common regulatory and professional settings, the actual implementation of the rules in practice can be different in hospitals. There is therefore a potential for uneven implementation to coincide with formal complying. This is particularly true for healthcare quality as failure is not due to the lack of standards but to low institutionalisation of coordination, accountability, learning and resource-management practices.

Kyambade *et al.* (2026) provide a recent African example of institutional theory, and show that institutional fragmentation, lack of policy coherence and limited accountability are responsible for the lack of sustainability of the health sector. Their focus is on health and sanitation projects, but the theoretical dimension pertains to the implementation of hospital services: organisational results depend on congruence between institutional rules, actors and routines. In the present study, HCDA and RAWS are considered as organisational learning and workforce capability, while SHP is considered as a mechanism and a process through which workforce capability is translated into service delivery.

### 2.3 Human capital development and organisational learning

Institutional activities that enhance knowledge, capability and the capacity to respond to the evolving needs of services are part of human capital development. Tacit knowledge can be related to experience in healthcare, but experience does not necessarily imply learning. Institutional theory suggests that learning has to be made more consequential via training, supervision, feedback, standard operating procedures and supportive norms if it is to become organisationally consequential. From a quality perspective, the Donabedian framework draws the same conclusion: workforce capacity is a structural input that has an impact on care processes.

The need for supportive practice environments for quality and patient safety has recently been demonstrated in South Africa's public hospitals (Tenza *et al.*, 2024). It is not only the number of years of service that should be used to evaluate human capital development but whether the organisational systems enable the staff to apply and update the human capital. This is a very strong hope that there will be a positive correlation between SHP and HCDA.

### 2.4 Research attitudes, work skills and service processes

RAWS is reflective of a broader attitude of the workforce – attitudes to inquiry, learning and work-related capability. While some of these attributes are individual, others are of an institutional nature and are therefore shaped by organisational rules and norms. Consistent service handling is more likely to be supported by routine development in hospitals which foster the use of evidence, reflection, learning and accountability. On the other hand, there are situations where, despite having good attitudes and skills, staff is not able to transfer it into good service quality due to strong organisational constraints.

This distinction is of paramount importance in a health system with scarce resources. Information management, inventory and facility-location dynamics are critical in the functioning of pharmaceutical distribution as shown by Mwakyeja and Kimario (2024), and digital pharmaceutical procurement processes can impact the continuity of health care supplies as illustrated by Mtengule *et al.* (2026). These studies give credence to the notion that individual ability is integral to operational systems. It is therefore important to view the use of RAWS as an enabling condition, and not as an independent service quality assurance.

### 2.5 Workforce experience

Years in the game is not always an indicator of ability; sometimes it is a misrepresentation. Experience can foster tacit knowledge and familiarity with hospital procedures and practices, but institutional inertia can also perpetuate ineffective practices. If professional development, supervision and process improvement are weak, there is only so much marginal improvement that can be made and more years of service means less marginal improvement. So the question that should be asked from the empirical point of view is not whether experience is intrinsically valuable or not, but whether the relationship between these service processes is large enough to be of practical significance.

The analysis in HCDA, SHP and RAWS can be utilised to explore this question. Reporting effect sizes along with statistical significance is important because a statistically detectable association may not be large enough to warrant decisions on policy based only on tenure.

### 2.6 Conceptual framework and hypotheses

The combination of two theories, namely the institutional theory and the process logic of Donabedian, has been used as the conceptual framework. Tenure is viewed as a pathway to organisational capability; the relationship between these three constructs is examined to see whether tenure is translated to organisational capability (HCDA and RAWS are viewed as institutional/workforce enabling conditions, SHP as the process-oriented service-quality outcome and work experience as an individual characteristic). The relations in the framework are not meant to be comprehensive of the institutional factors that determine the quality, but rather a subset of relations that can be explored using the variables available.

H1.
Human Capital Development Activities (HCDA) is positively related to Service Handling Practices (SHP) to the extent counselling skills.

H2.
There is a positive relationship between H2: Research Attitudes and Work Skills (RAWS) and SHP.

H3.
There is a relationship between work experience and HCDA.

H4.
Work experience is related to SHP.

H5.
There is a relationship between work experience and RAWS.

## 3. Methodology

### 3.1 Research design

A cross-sectional survey design with a quantitative approach was adopted. This model can be used as an approximation when an approximation of relations between observed organisational and workforce variables at a particular moment in time is desired, but causality and ordering of variables can only be approximated. It is explanatory rather than causal and estimates the strengths of the relationships and then interprets them in the light of institutional theory and the Donabedian model.

### 3.2 Study setting and unit of enquiry

This was a study in selected state-owned public hospitals in KwaZulu-Natal, South Africa. The province was chosen because it has a public hospital system that has public hospitals that cater to urban and rural populations as well as different levels of referral care. The provincial planning documents outline a network of public hospitals, with service delivery, quality and system performance consistently being a focus of attention (KwaZulu-Natal Department of Health, 2025).

The respondents of the unit of enquiry were individuals from hospitals that participated in or were directly involved in service-delivery processes. The study was aimed at employees in the hospital and those who use health services in the hospital. The analytic file received was de-identified, and no reliable profession-specific codes (e.g. nurse, medical doctor or pharmacist) were provided, nor was a facility identifier provided for validation in multilevel analysis. Hence, no statistical claims are made that are specific to any profession or hospital and for which there is no data. This is not an implicit, but an explicit limitation on professional categories from unlabelled codes.

### 3.3 Sampling and sample size

A multistage procedure was used during fieldwork. Hospitals were purposively selected for relevance and accessibility, after which respondents were recruited within the selected institutions using systematic and convenience-based procedures according to operational access and willingness to participate. These procedures limit probability-based generalisation but are consistent with the practical constraints of hospital-based survey research.

A total of 238 questionnaires were available in the respondent-level dataset. Four cases contained an isolated missing item across one or more scales; consequently, 234 complete cases were used for the multivariable regression. For a two-predictor regression at α = 0.05, *N* = 234 provides approximately 80% power to detect an incremental effect of about f^2^ = 0.042 (equivalent to *R*^2^ of approximately 0.040), based on the noncentral-F approach underlying conventional regression power analysis (Faul *et al.*, 2009). The sample is therefore adequate for detecting small-to-moderate overall regression effects, although power does not remedy measurement or sampling limitations.

### 3.4 Research instrument and variables

Data were collected using a structured questionnaire with five-point Likert-type response options. The analysis uses the three-item blocks that can be directly verified in the data file: HCDA (12 items), SHP (9 items) and RAWS (13 items). Scale scores were calculated as the respondent's mean across available items within each block; the multivariable model used complete cases across all three item blocks.

SHP is used as the dependent variable because it is the available construct most directly concerned with the process of handling services. It should therefore be interpreted as a process-oriented indicator of perceived service quality, not as a comprehensive measure of clinical quality or patient outcomes. HCDA and RAWS are used as predictors representing institutional learning/workforce development and workforce attitudes/skills, respectively. Work experience was used for the group-comparison analysis (see Figure 1).

**Figure 1:**
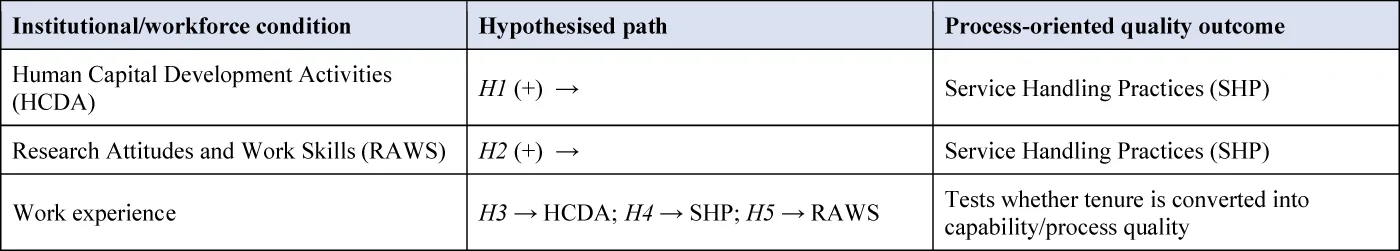
Conceptual framework and hypothesis paths relationships are hypothetical and shown with arrows. HCDA and RAWS are conditions that allow a process-based service quality outcome to be realised: SHP

### 3.5 Construct validity and reliability

Construct adequacy was assessed from the respondent-level dataset. Kaiser–Meyer–Olkin (KMO) statistics and Bartlett's tests of sphericity were used to determine whether item correlations were suitable for exploratory component analysis. Cronbach's alpha was calculated from complete item responses within each scale. Because the item blocks showed more than one eigenvalue above 1, the analyses are interpreted as evidence of multidimensionality rather than as confirmation of three strictly unidimensional latent variables. The composite scores are therefore treated as broad empirical indices, and this measurement limitation is carried into the interpretation of the regression.

HCDA showed good internal consistency (α = 0.829) and acceptable sampling adequacy (KMO = 0.837; Bartlett χ^2^(66) = 2299.80, *p* < 0.001). SHP showed lower internal consistency (α = 0.648) and marginal KMO adequacy (KMO = 0.557; Bartlett χ^2^(36) = 1385.33, *p* < 0.001), indicating that the nine SHP items should be interpreted cautiously as a broad process index. RAWS showed acceptable internal consistency (α = 0.780) and KMO = 0.796, with Bartlett χ^2^(78) = 1217.58, *p* < 0.001. These findings offer a clear construct check and illustrate the reasons why statements of the existence of a single service quality factor should be eschewed (see Table 1)

**Table 1:** Construct adequacy and reliability from the respondent-level dataset

Construct	Items	Complete n	Cronbach α	KMO	Bartlett χ^2^ (df)	*p*	Eigenvalues >1
HCDA	12	237	0.829	0.837	2299.80 (66)	<0.001	2
SHP	9	237	0.648	0.557	1385.33 (36)	<0.001	4
RAWS	13	236	0.780	0.796	1217.58 (78)	<0.001	4

**Note(s):** KMO = Kaiser–Meyer–Olkin measure. If there is more than one eigenvalue greater than 1, one can infer the presence of multidimensionality and composites are not considered as unidimensional latent variables

### 3.6 Data collection and ethics

The questionnaires were auto-administered in the selected hospitals. This was voluntary participation; informed consent was obtained, and confidentiality and anonymity were preserved. Data collection was done after obtaining ethical approval from the proper institutional source. No identifying participant information is reported in the manuscript, and the dataset used for the analysis was de-identified.

### 3.7 Data analysis and statistical assumptions

Descriptive statistics were used to summarise respondent characteristics and the three composite scales. The available one-way ANOVA output was used to examine differences across work-experience groups for HCDA, SHP and RAWS, with eta-squared and omega-squared reported to distinguish statistical from practical significance. The work-experience grouping variable was not present in the respondent-level spreadsheet used for the regression analysis, so the available ANOVA F statistics and effect-size estimates were reported, but the homogeneity test could not be independently reproduced. For that reason, ANOVA results are treated as secondary evidence and interpreted cautiously.

Multiple regression specified SHP as the dependent variable, with HCDA and RAWS entered simultaneously as predictors. Multicollinearity was assessed using tolerance and variance inflation factors (VIFs), and residual independence using the Durbin–Watson statistic. VIF was 1.40 for both predictors (tolerance = 0.716) and Durbin–Watson was 2.06, indicating no material multicollinearity or residual autocorrelation. The Breusch–Pagan test indicated heteroscedasticity (*p* < 0.001), while residual normality tests also indicated departure from normality. Therefore, HC3 heteroscedasticity-consistent standard errors and confidence intervals are reported for coefficient inference. The overall model F test and *R*^2^ are reported as model-fit descriptors, but the results are interpreted conservatively given the diagnostic findings and bounded Likert-type outcome.

## 4. Results

### 4.1 Respondent profile

The respondent profile shows that 189 participants (79.4%) reported matric as their highest educational qualification, 26 (10.9%) a diploma, 17 (7.1%) ABET, five (2.1%) a degree and one (0.4%) a postgraduate qualification. The available work-experience output showed that 70.6% of respondents were concentrated between 10 and 20 years of experience, with 38.2% reporting 10–15 years and 32.4% reporting 16–20 years (see Table 2). These distributions provide context for interpreting the limited effect of tenure identified below.

**Table 2:** Educational qualification of respondents (*N* = 238)

Qualification	*n*	%
ABET	17	7.1
Matric	189	79.4
Diploma	26	10.9
Degree	5	2.1
Postgraduate	1	0.4
Total	238	100.0

### 4.2 Work-experience distribution

The respondents' work experience shows that 91 (38.2%) had between 10 and 15 years of experience, representing the largest group. This was followed by 77 respondents (32.4%) with 16–20 years of experience and 34 (14.3%) with 21–25 years. A smaller number, 11 respondents (4.6%), had 4–9 years of experience, while only five (2.1%) had 30 years or more of experience (see Table 3). These findings show that most respondents had considerable work experience, particularly between 10 and 20 years.

**Table 3:** Work-experience distribution (*N* = 238)

Years of experience	*n*	%
0–3	8	3.4
4–9	11	4.6
10–15	91	38.2
16–20	77	32.4
21–25	34	14.3
26–29	12	5.0
30 and above	5	2.1
Total	238	100.0

### 4.3 Descriptive statistics and correlations

Among the 234 complete cases used for regression, mean HCDA was 2.80 (SD = 0.61), mean SHP was 3.39 (SD = 0.51) and mean RAWS was 2.71 (SD = 0.47). HCDA and SHP were positively correlated (*r* = 0.160, *p* = 0.014), RAWS and SHP were positively correlated (*r* = 0.173, *p* = 0.008), and HCDA and RAWS showed a moderate positive correlation (*r* = 0.533, *p* < 0.001). The latter correlation supports the conceptual argument that human-capital development and workforce attitudes/skills are related aspects of institutional capability, while the low VIF confirms that their overlap is not severe enough to destabilise the regression model (see Table 4)

**Table 4:** Descriptive statistics and Pearson correlations for regression complete cases (*N* = 234)

Variable	Mean	SD	1	2	3
1. HCDA	2.799	0.612	1.000		
2. SHP	3.389	0.515	0.160*	1.000	
3. RAWS	2.715	0.468	0.533***	0.173**	1.000

**Note(s):** *p < 0.05; **p < 0.01; ***p < 0.001 (two-tailed)

### 4.4 Work-experience differences

The available one-way ANOVA results show a statistically significant difference in HCDA across work-experience groups (F = 2.221, *p* = 0.042), but the omega-squared effect size was only 0.030, indicating a small practical effect. Differences were not statistically significant for SHP (F = 1.212, *p* = 0.234) or RAWS (F = 0.645, *p* = 0.659). Their omega-squared estimates were 0.009 and −0.008, respectively, indicating very small or negligible practical effects. Thus, the data support an association between tenure and some human-capital development activity, but not a general claim that longer experience improves service handling or work-related attitudes/skills (see Table 5).

**Table 5:** ANOVA results and effect sizes by work-experience group

Outcome	F	*p*	η^2^	ω^2^	Interpretation
HCDA	2.221	0.042	0.055	0.030	Significant; small
SHP	1.212	0.234	0.034	0.009	Not significant; very small
RAWS	0.645	0.659	0.018	−0.008	Not significant; negligible

**Note(s):** The work-experience grouping variable was not present in the spreadsheet used for regression analysis; therefore, the available ANOVA output is reported as secondary evidence

### 4.5 Multiple regression analysis

The model regressed SHP on HCDA and RAWS using 234 complete cases. It was statistically significant overall, F(2,231) = 4.346, *p* = 0.014, with *R* = 0.190, *R*^2^ = 0.036 and adjusted *R*^2^ = 0.028. The model therefore explains approximately 3.6% of the observed variance in SHP, indicating modest explanatory power (see Table 6).

**Table 6:** Regression model summary and diagnostics

Statistic	Value
*N*	234
*R*	0.190
*R* ^2^	0.036
Adjusted *R*^2^	0.028
F (df1, df2)	4.346 (2, 231)
Model p	0.014
Durbin–Watson	2.062
VIF (both predictors)	1.397
Tolerance	0.716
Breusch-Pagan p	<0.001; HC3 robust SE used

Using HC3 robust standard errors, HCDA had a positive but non-significant adjusted association with SHP (B = 0.080, robust SE = 0.090, β = 0.095, *t* = 0.887, *p* = 0.376, 95% CI [−0.098, 0.258]). RAWS also had a positive but non-significant adjusted association (B = 0.134, robust SE = 0.093, β = 0.122, *t* = 1.438, *p* = 0.152, 95% CI [−0.050, 0.318]). Accordingly, H1 and H2 are not supported at the 5% level in the multivariable model. The finding is important because it prevents overstatement: the two institutional/workforce indices are related to service handling at the bivariate level, but neither independently accounts for a large share of variation once their overlap is considered (see Table 7).

**Table 7:** Multiple regression predicting SHP (HC3 robust inference)

Predictor	B	HC3 SE	β	*t*	*p*	95% CI for B
Constant	2.800	0.233	–	12.011	<0.001	[2.341, 3.260]
HCDA	0.080	0.090	0.095	0.887	0.376	[−0.098, 0.258]
RAWS	0.134	0.093	0.122	1.438	0.152	[−0.050, 0.318]

**Note(s):** Dependent variable: SHP. β = standardised coefficient. HC3 standard errors are reported because the Breusch-Pagan test indicated heteroscedasticity

### 4.6 Hypothesis summary

The hypothesis tests indicate a mixed pattern. H1 and H2 are not supported by the robust multiple regression. H3 is supported statistically but with a small effect size. H4 and H5 are not supported. The pattern is consistent with an institutional explanation in which service processes are shaped by a wider configuration of governance, resources, coordination and routines than the measured workforce indices alone (see Table 8).

**Table 8:** Summary of hypothesis tests

Hypothesis	Test	Result	Decision
H1: HCDA is positively associated with SHP	Robust multiple regression	β = 0.095, *p* = 0.376	Not supported
H2: RAWS is positively associated with SHP	Robust multiple regression	β = 0.122, *p* = 0.152	Not supported
H3: Work experience is associated with HCDA	One-way ANOVA	F = 2.221, *p* = 0.042; ω^2^ = 0.030	Supported, small effect
H4: Work experience is associated with SHP	One-way ANOVA	F = 1.212, *p* = 0.234	Not supported
H5: Work experience is associated with RAWS	One-way ANOVA	F = 0.645, *p* = 0.659	Not supported

## 5. Discussion

The results challenge single-determinant explanations of service quality. HCDA and RAWS are positively related to service handling at the bivariate level, but they explain only a small portion of SHP variation and are not independently significant under heteroskedasticity-robust inference. This pattern indicates that workforce development and work skills are enabling conditions rather than sufficient explanations of service handling. The study's contribution therefore lies in showing why institutional context and organisational processes matter alongside individual workforce attributes.

Institutional theory helps explain this result. Formal learning, workforce competencies and positive attitudes are facilitating factors, but if they do not lead to positive practice, the organisational rules, routines and expectations will not (Scott, 2014). Despite building a human capital, a hospital can still be facing the patient flow problem, coordination issues, supply chain issues, lack of accountability and lack of supervision. In this case, the competency of the staff might not directly relate to the handling of the services. This is consistent with what Kyambade *et al.* (2026) have found: fragmented governance and weak institutional alignment can have a detrimental effect on health-sector performance, even with policy support.

A complementary interpretation is offered by the Donabedian model. HCDA and RAWS are more a structural/organisational measure, SHP is a process measure. Adjusted effects are weak, suggesting that the capacity to process is not automatic or complete. Much of the variation that remains is likely to be the result of other structural factors and process mechanisms, such as staffing levels, leadership, workflow, availability of equipment, reliability of procurement, information systems and patient demand. The low *R*^2^ value is thus not only disappointing, but also substantively informative, in the sense that it warns about a limited view of the quality of a service based on a few measures of the workforce.

This is confirmed by the work-experience results. Tenure related to HCDA, but the effect was not great and there were no significant differences in SHP or RAWS. Experience can help build up knowledge, but institutional learning needs mechanisms to refresh and codify knowledge and to apply it. Stable routines over the years, with no ongoing PD, feedback and supportive supervision, do not enhance the way services are delivered. This is similar to South African results, which indicated that quality and safety go hand in hand and are not only influenced by the individual worker's characteristics (Tenza *et al.*, 2024).

The findings also connect with African operational research beyond workforce management. Mwakyeja and Kimario (2024) demonstrate the importance of information, inventory and distribution dynamics in pharmaceutical performance, while Mtengule *et al.* (2026) show that digital procurement arrangements can change the resilience of healthcare supply processes. These studies illustrate institutional mechanisms that are not captured by HCDA or RAWS but can directly shape hospital service reliability. A stronger future model of public-hospital quality should therefore connect workforce development with governance, procurement, infrastructure, information and patient-flow measures.

At the South African level, Babalola *et al.* (2022) found substantial variation in technical efficiency across KwaZulu-Natal district hospitals and linked efficiency to utilisation, staffing and expenditure factors. The present study does not reproduce a hospital-level efficiency analysis because reliable facility identifiers were not available in the de-identified dataset. Nevertheless, the two studies converge on an important point: institutional performance is multi-causal. The correct policy response is therefore to strengthen the organisational system through which resources and staff capability are converted into quality, rather than assuming that additional tenure, infrastructure or training will have uniform effects in isolation.

Finally, the theoretical reframing strengthens the international relevance of the study. SDG 3.8 treats access to quality essential healthcare services as part of universal health coverage, while AU Agenda 2063 Goal 3 explicitly links continental development to expanded access to quality healthcare. The present results suggest that achieving these ambitions requires attention to institutional reliability: how hospitals coordinate learning, people, resources and patient-facing processes. The study thus provides an empirical message of caution which has a wider applicability for resource-poor systems: quality improvement should focus on institutional arrangements rather than on individual inputs.

## 6. Implications

### 6.1 Theoretical implications

The study is a theoretical one because it is a mixture of institutional theory and Donabedian's structure-process-outcome approach. Both institutional theory help to account for the diversity of local practice from common policies and formal standards and Donabedian to understand the mechanism by which organisational capacity has to be translated into care processes before it can impact outcomes. This combined view is supported by the low direct regression effects; the capability of the workforce is a necessary but not sufficient precondition, and there is a need for explicit modelling of organisational mechanisms that connect workforce capability with handling services.

The results also caution against treating composite survey variables as self-contained causal determinants. Construct analyses show multidimensionality, particularly for SHP and RAWS. Future theoretical models should distinguish governance, organisational learning, work climate, communication, infrastructure and service processes as separate but interacting institutional domains, followed by confirmatory measurement before testing structural relationships.

### 6.2 Managerial and practical implications

Hospital managers should not use length of service as a proxy for service-quality capability. Professional development should be linked to supervision, standardised workflows, feedback, communication and performance review. HCDA initiatives are most likely to influence service handling when they are embedded in day-to-day routines and reinforced by managers.

Second, service-quality monitoring should move beyond staff demographics. Management dashboards should combine workforce development with patient-flow indicators, complaint resolution, waiting times, communication quality, medicine and equipment availability, incident reporting and patient experience. Third, where diagnostic data indicate that one broad scale contains multiple dimensions, managers should avoid relying on a single aggregate score and should monitor the underlying service processes separately.

### 6.3 Policy and societal implications

For provincial and national policymakers, the results support quality-improvement strategies that combine human-resource development with governance and operational reform. This involves secure information systems, procurement and supply-chain management, accountability systems and practice environments and regular systems of patient-experience measurement. This is in line with SDG 3.8 and Agenda 2063 Goal 3 that calls for access to quality care and not only care.

The social aspect is not to be discounted. Waiting, communication, reliability, dignity and continuity are elements of institutional quality that are experienced by patients, along with clinical treatment. Thus, the organisation of the supporting structures and processes for frontline care can improve public confidence, and public hospital user experience, even in the face of constrained resources.

## 7. Limitations and future research

There are some limitations to be taken into consideration when interpreting. First, the cross-sectional design helps to find associations and not causal effects. Second, data were self-reported and there may be common method or social desirability bias. Third, sampling using convenience and purposive and systematic sampling prevents generalisation beyond the sampling contexts, in terms of probability. Fourth, the analytic file supplied for revision was not validated and hence did not match a verified hospital identifier or profession-specific labels and the study does not provide a valid estimate of the hospital-level random effects or a valid comparison of different professions such as nurses, doctors, pharmacists, etc. Without risk of misclassification.

Fifth, SHP is a process-oriented index, not a clinical-quality outcome. The construct analyses show that the item blocks are multidimensional; SHP in particular has only marginal internal consistency (α = 0.648) and KMO adequacy (0.557). This limits the precision with which the regression can represent a single service-quality construct. Sixth, the regression explains only 3.6% of SHP variance and exhibits heteroscedasticity and non-normal residuals; robust standard errors reduce the risk of incorrect coefficient inference but do not remove model misspecification. Seventh, the supplied respondent-level spreadsheet did not contain the work-experience group variable, so the original ANOVA assumption tests could not be independently reproduced and those results are therefore treated as secondary.

Future research should collect hospital identifiers and profession-specific categories prospectively and use stratified probability sampling where feasible. Confirmatory factor analysis should validate the measurement structure before hypothesis testing. Multilevel longitudinal models could then distinguish individual, professional and hospital-level effects and examine whether changes in governance, training, procurement or workflow precede changes in patient experience and clinical outcomes. Future models should also incorporate objective indicators such as waiting time, complaint resolution, medicine availability, staffing intensity, adverse events and readmissions. Comparative work across South African provinces and other African health systems would allow the institutional explanation proposed here to be tested beyond KwaZulu-Natal.

## 8. Conclusion

This study explains healthcare service quality in selected KwaZulu-Natal public hospitals by aligning the empirical model with variables that can be verified in the respondent-level dataset and by grounding interpretation in institutional theory and Donabedian's quality framework. HCDA and RAWS are positively related to service handling at the bivariate level, but the regression shows that their independent effects are small and statistically uncertain. Work experience is associated with HCDA only weakly in practical terms and does not differentiate service handling or RAWS.

The central conclusion is therefore not that a single resource or workforce characteristic determines hospital quality. Service quality is an institutional accomplishment: staff capability must be converted into reliable service processes through governance, coordination, learning, information, resource management and accountability. This explanation provides a more defensible basis for management and policy action and aligns the study with the quality-of-care ambitions of SDG 3 and AU Agenda 2063.

## Data Availability

The de-identified data supporting the statistical analysis are available from the corresponding author subject to applicable ethical and institutional requirements.
